# Evolutionary history predicts high‐impact invasions by herbivorous insects

**DOI:** 10.1002/ece3.5709

**Published:** 2019-10-17

**Authors:** Angela M. Mech, Kathryn A. Thomas, Travis D. Marsico, Daniel A. Herms, Craig R. Allen, Matthew P. Ayres, Kamal J. K. Gandhi, Jessica Gurevitch, Nathan P. Havill, Ruth A. Hufbauer, Andrew M. Liebhold, Kenneth F. Raffa, Ashley N. Schulz, Daniel R. Uden, Patrick C. Tobin

**Affiliations:** ^1^ School of Environmental and Forest Sciences University of Washington Seattle Washington; ^2^ Southwest Biological Science Center U.S. Geological Survey Tucson Arizona; ^3^ Department of Biological Sciences Arkansas State University Jonesboro Arkansas; ^4^ The Davey Tree Expert Company Kent Ohio; ^5^ Nebraska Cooperative Fish and Wildlife Unit School of Natural Resources U.S. Geological Survey University of Nebraska‐Lincoln Lincoln Nebraska; ^6^ Department of Biological Sciences Dartmouth College Hanover New Hampshire; ^7^ D.B. Warnell School of Forestry and Natural Resources University of Georgia Athens Georgia; ^8^ Department of Ecology and Evolution Stony Brook University Stony Brook New York; ^9^ Northern Research Station USDA Forest Service Hamden Connecticut; ^10^ Department of Bioagricultural Science and Pest Management Colorado State University Fort Collins Colorado; ^11^ USDA Forest Service Northern Research Station Morgantown West Virginia; ^12^ Department of Entomology University of Wisconsin Madison Wisconsin; ^13^ Nebraska Cooperative Fish and Wildlife Unit Department of Agronomy and Horticulture School of Natural Resources University of Nebraska‐Lincoln Lincoln Nebraska

**Keywords:** evolutionary history, herbivore, invasive insect, non‐native species, risk assessment

## Abstract

1. A long‐standing goal of invasion biology is to identify factors driving highly variable impacts of non‐native species. Although hypotheses exist that emphasize the role of evolutionary history (e.g., enemy release hypothesis & defense‐free space hypothesis), predicting the impact of non‐native herbivorous insects has eluded scientists for over a century.
2. Using a census of all 58 non‐native conifer‐specialist insects in North America, we quantified the contribution of over 25 factors that could affect the impact they have on their novel hosts, including insect traits (fecundity, voltinism, native range, etc.), host traits (shade tolerance, growth rate, wood density, etc.), and evolutionary relationships (between native and novel hosts and insects).
3. We discovered that divergence times between native and novel hosts, the shade and drought tolerance of the novel host, and the presence of a coevolved congener on a shared host, were more predictive of impact than the traits of the invading insect. These factors built upon each other to strengthen our ability to predict the risk of a non‐native insect becoming invasive. This research is the first to empirically support historically assumed hypotheses about the importance of evolutionary history as a major driver of impact of non‐native herbivorous insects.
4. Our novel, integrated model predicts whether a non‐native insect not yet present in North America will have a one in 6.5 to a one in 2,858 chance of causing widespread mortality of a conifer species if established (*R* ^2^ = 0.91)
5. *Synthesis and applications*. With this advancement, the risk to other conifer host species and regions can be assessed, and regulatory and pest management efforts can be more efficiently prioritized.

## Graphical abstract

This research is the first to empirically support historically assumed hypotheses about the importance of evolutionary history as a major driver of impact of non‐native herbivorous insects. We discovered that evolutionary history between native and novel hosts, life history traits of the novel host, and the evolutionary relationship between non‐native and coevolved insects on a shared host were more predictive of impact than traits of the invading insect. Our novel, integrated model predicts whether a non‐native insect not yet present in North America will have a one in 6.5 to a one in 2,858 chance of causing widespread mortality of a conifer species if established.

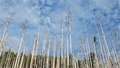

## 1 INTRODUCTION

**Figure 1:**
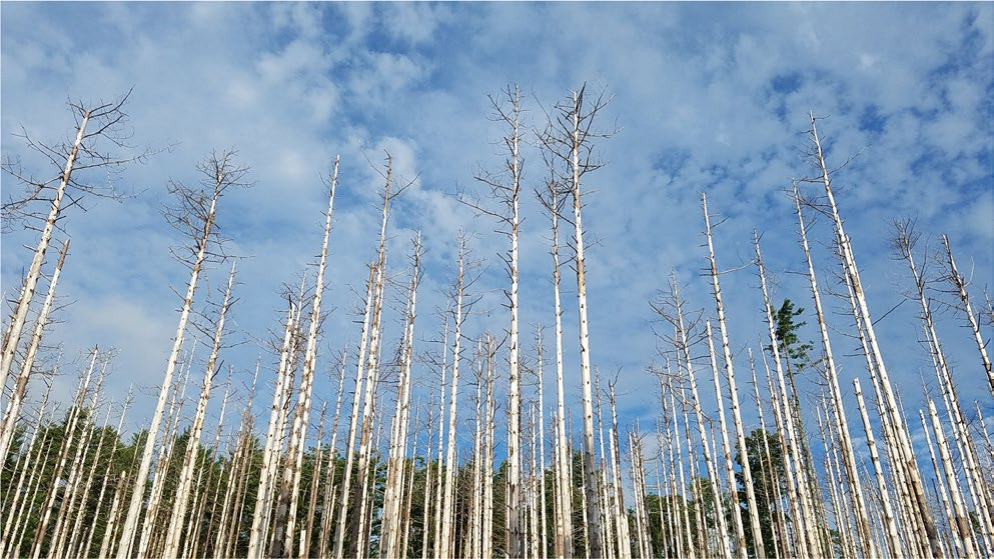
Example of high‐impact damage caused by a non‐native insect: Red pines (*Pinus resinosa*) killed by the red pine scale (*Matsucoccus matsumurae*) near Myles Standish State Forest, Massachusetts. Photograph by Jeff Garnas, University of New Hampshire

Expansion of global trade has increased establishment of non‐native herbivorous insects (Aukema et al., 2010), most of which cause a little impact in their invaded range (Williamson & Fitter, 1996). A small minority of invaders, however, cause high impacts that exceed US$70 billion annually just in North America (Bradshaw et al., 2016), making it imperative to predict which species pose the greatest risk. We define high‐impact species as those that cause mortality of their host plants at population or regional scales, disrupting ecological systems, and causing serious environmental or socioeconomic harm (Figure 1). Although there have been advances in the ability to predict the establishment of non‐native invaders (Gallien, Thornhill, Zurell, Miller, & Richardson, 2019), identifying predictors of impact once they have established has proven difficult (Kolar & Lodge, 2001).

A long‐held assumption regarding the success of non‐native invaders relates to the absence of their coevolved natural enemies in the introduced range (enemy release hypothesis; Keane & Crawley, 2002), which has motivated classical biological control programs against non‐native herbivorous insects for 130 years (Burgess & Crossman, 1929; Caltagirone, 1981). Similarly, the defense‐free space hypothesis invokes lack of coevolved host defenses as a factor responsible for high‐impact herbivore invasions (Gandhi & Herms, 2010). Although the success of some classical biological control programs provides empirical support for the enemy release hypothesis (DeBach & Rosen, 1991), and a lack of coevolved defenses against some invasive herbivorous insects has been documented (Brooks, Ervin, Varone, & Logarzo, 2012; Desurmont, Donoghue, Clement, & Agrawal, 2011; Woodard, Ervin, & Marsico, 2012), these hypotheses have not been applied to predict the impact of non‐native insects. Recent frameworks have integrated multiple, single‐factor hypotheses into synthetic theories of invasion success (e.g., Barney & Whitlow, 2008; Catford, Jansson, & Nilsson, 2009), but these are too general for making specific predictions and may mask important mechanisms driving the impact of invasions. Simultaneous consideration of multiple traits of non‐native insects and their hosts may better predict the probability of high‐impact invasions (e.g., Gurevitch, Fox, Wardle, Inderjit, & Taub, 2011).

We tested the hypothesis that multiple traits better predict high‐impact invasions by focusing on non‐native insect herbivores in North America that specialize on coniferous (Order Pinales) trees (hereafter, conifer specialists), which are widely distributed across latitude and elevation, dominate multiple biomes, are well studied, and have great ecological and economic value (Eckenwalder, 2009). Specifically, we tested if the probability of a non‐native conifer specialist causing high impact on a North American (novel) conifer host is a function of the following: (a) evolutionary divergence time between the native and novel hosts, (b) life history traits of its novel host, especially those traits related to herbivore resistance and tolerance, (c) the evolutionary relationship of the non‐native conifer specialist to native insects that have coevolved with the shared North American host, and/or (d) the life history traits of the non‐native insect. We quantified the contributions of these factors, individually and in combination, to assess the magnitude of impact and provide a model framework for predicting which introduced insect herbivores are likely to be high‐impact invaders. We also demonstrate that a composite model substantially increases predictability relative to individual submodels. Our research is the first to generate quantitative evidence for the role of evolutionary history as a predictor of the impact of non‐native insect herbivores on their host plants.

## 2 MATERIALS AND METHODS

### 2.1 Insect traits

We censused non‐native insects, using published lists (Aukema et al., 2010; Yamanaka et al., 2015), and identified 58 conifer specialists currently established in North America that feed on hosts in Cupressaceae, Pinaceae, and/or Taxaceae (Table A1). For each conifer specialist, literature and online searches were conducted (March 2016–July 2017) to find: (a) values of 15 potentially relevant insect life history traits including fecundity and voltinism, (b) the highest level of plant damage described in published literature, (c) all documented North American host trees (excluding conifers outside their native range in North America), and (d) all host trees from the insect's native range. High‐impact insects were defined as those reported to cause tree mortality at the population or regional level (Figure 1), whereas species that directly or indirectly caused only individual tree mortality or minor damage were not considered to be high impact (Table 1). A binary impact response variable was considered useful for decision‐making (high impact or not), avoided the subjectivity of multiple impact levels, and eliminated the potential effect of time since introduction. For example, a recently introduced species with a limited distribution would qualify as high impact if it had caused mortality in its localized host population, recognizing that it could potentially spread over time.

**Table 1:** Description of documented non‐native insect impacts on naïve hosts, independent of management programs

Impact number	High impact	Description
1	0	No damage documented in the literature.
2	0	Minor damage; examples: leaf/needle loss, leaf/needle discoloration, twig dieback, or fruit drop.
3	0	Mortality of individual stressed plants.
4	0	Weakening of an individual plant that suffers mortality from another agent.
5	0	Mortality of individual healthy plants.
6	1	Isolated or sporadic mortality within an affected plant population^a^; examples: occasional outbreaks that yield > 10% mortality, 90% mortality with regeneration, or sustained mortality of 5% per year in multiple populations.
7	1	Extensive or persistent mortality within a population; example: more than 25% mortality over 10 years.
8	1	Wave of plant mortality with regional spread of the insect.
9	1	Functional extinction of the host plant.

Note

Binomial high‐impact value: 1 = yes; 0 = no.

a A population is defined as a spatially continuous group of interbreeding individuals.

Information available about non‐native conifer specialists in North America is concentrated on species causing the most damage. Hence, some insect traits, such as fecundity, were unavailable for many species and were not included in the analyses. Further, there were strong associations between insect order and feeding guild, as well as between the number of native host genera and degree of host specificity in the native range; thus, these pairs were reduced to a single trait (feeding guild and number of native host genera, respectively) for analyses. Eight insect traits were ultimately evaluated as predictors of impact (Table 2). We used multimodel inference within an information theoretic framework (Burnham & Anderson, 2003) to rank 12 unique generalized linear models (GLM; Table 2). Candidate models included the null (no predictors) and global (all predictors). Models were ranked based on Akaike's Information Criteria adjusted for small sample size (AICc). AICc scores and weights were calculated with the *GLM* and *AICTAB* functions in the stats and AICcmodavg packages for R, respectively (Mazerolle, 2019; R Core Team, 2017).

**Table 2:** Ranking of alternative models explaining variability in high‐impact insect invasions on North American conifers as a function of non‐native insect traits

Model	*K*	AICc	ΔAICc	*w*
Voltinism	2	43.308	**0.000**	0.27
Voltinism + Reproductive Strategy + Dispersal	5	43.911	**0.603**	0.20
Reproductive Strategy	2	44.475	**1.168**	0.15
Null Model	1	44.794	**1.486**	0.13
Congener	2	46.073	2.765	0.07
Number of Genera	2	46.305	2.997	0.06
Pest Status	2	46.733	3.426	0.05
Dispersal	2	46.791	3.483	0.05
Native Range	3	48.339	5.031	0.02
Guild	4	50.651	7.343	0.01
Native Range + Pest Status + Number Genera	5	51.935	8.627	<0.01
Global model	11	64.639	21.331	<0.01

Note

Lower Akaike's Information Criterion adjusted for small sample size (AICc) scores and higher AICc weights (*w*) indicate a greater relative degree of support for the model from the data. *K* indicates the number of parameters in each model, and ΔAICc is used to facilitate comparisons between the best‐supported model (AICc = 0.00) and other models. All models with ΔAICc scores ≤ 2.00 (bold font) were included in the confidence set.

### 2.2 Host traits

Our literature review revealed 49 North American conifer species that were fed upon by the 58 conifer specialists (Table A2). Six traits (foliage texture, growth rate, drought tolerance, fire tolerance, shade tolerance, and wood density) conceptually relevant to host quality were extracted for each conifer species from three sources: the United States Department of Agriculture Plants Database (USDA & NRCS, 2016); the TRY Database (Kattge et al., 2011); and Miles and Smith (2009); foliar carbon–nitrogen ratio and specific leaf area data were unavailable for many conifers and were therefore not included. As with insect traits, we used multimodel inference to evaluate 10 candidate models (Table 3) that related host traits with the probability of high impact for each novel insect–host pair (*n* = 221).

**Table 3:** Ranking of alternative models explaining variability in high‐impact insect invasions as a function of host tree traits

Model	*K*	AICc	ΔAICc	*w*
Shade tolerance + Drought tolerance	6	109.547	**0.000**	0.79
Growth rate	3	114.765	5.218	0.06
Wood density + Growth rate	4	114.929	5.382	0.05
Wood density	2	115.567	6.020	0.04
Null model	1	116.849	7.302	0.02
Foliage texture + Growth rate	5	116.863	7.317	0.02
Foliage texture	3	118.605	9.058	<0.01
Drought tolerance	4	119.142	9.595	<0.01
Global model	14	121.842	12.295	<0.01
Fire tolerance + Drought tolerance	7	124.834	15.287	<0.01

Note

Lower Akaike's Information Criterion adjusted for small sample size (AICc) scores and higher AICc weights (*w*) indicate a greater relative degree of support for the model from the data. *K* indicates the number of parameters in each model, and ΔAICc is used to facilitate comparisons between the best‐supported model (AICc = 0.00) and other models. All models with ΔAICc scores ≤ 2.00 (bold font) were included in the confidence set.

### 2.3 Host evolutionary history

Each insect–host pair was matched with each coevolved (native) host of the insect in its native range (*n* = 1,271 triplets). Divergence time (millions of years ago; mya) between the novel and native host was assigned for each triplet using the nearly comprehensive dated phylogeny of conifers by Leslie et al. (2012). For three species not represented in this phylogeny (*Abies balsamea* (L.) Miller, *Pinus cembra* L., and *P. banksiana* Lambert), divergence times were inferred using dates among clades in Leslie et al. (2012) and their positions in other published phylogenies (Gernandt, López, Garcia, & Liston, 2005; Parks, Cronn, & Liston, 2012; Xiang et al., 2015). For each triplet, the distance to the most recently diverged host in the insect's native range was extracted for analyses, which minimized the impact of incomplete host records and ensured independence among observations. Three pairs were excluded because the globally distributed *Juniperus communis* L. was both the North American and closest native Eurasian host, leaving 218 pairs. Using logistic regression and the chi‐squared likelihood ratio (*G* ^2^), we tested for effects of divergence time between the closest native and novel host plants, feeding guild of the insect, and interaction between the two, on the probability of high impact. Since there was a strong interaction term, we tested separate models for each feeding guild. Visual examination of the data suggested nonlinearities between divergence time and impact; thus, we also considered models that included a squared term for divergence time (RMS package; Harrell, 2017).

### 2.4 Insect evolutionary history

Sharing a host with a closely related herbivore native to North America could influence the impact of an invading non‐native insect. To test this hypothesis, we compiled a list of North American insect genera associated with each North American conifer in our analyses using the following sources: Blackman and Eastop (1994), Burns and Honkala (1990), Drooz (1985), Furniss and Carolin (1977), Johnson and Lyon (1991), Pickering (2011), Robinson, Ackery, Kitching, Baccaloni, and Hernández (2010), and Wood and Bright (1992). To account for false negatives generated by any undocumented native insect genera, we excluded the 10% of conifers (*n* = 8) with the fewest documented insect genera. For the remaining 203 insect–host pairs, we evaluated models predicting the probability of high impact based on the presence or absence, on the same host, of a co‐occurring native insect in the same genus or family as the non‐native conifer specialist (Table 4). However, we did not evaluate the global model because insects in the same genus are also in the same family.

**Table 4:** Ranking of alternative models explaining variability in high‐impact insect invasions as a function of the taxonomic relationship between non‐native conifer specialists and their closest North American insect relative on the same host tree species

Model	*K*	AICc	ΔAICc	*w*
Shared genus	2	98.778	**0.000**	0.89
Null model	1	103.908	5.129	0.07
Shared family	2	104.958	6.179	0.04

Note

Lower Akaike's Information Criterion adjusted for small sample size (AICc) scores and higher AICc weights (*w*) indicate a greater relative degree of support for the model from the data. *K* indicates the number of parameters in each model, and ΔAICc is used to facilitate comparisons between the best‐supported model (AICc = 0.00) and other models. All models with ΔAICc scores ≤ 2.00 (bold font) were included in the confidence set.

### 2.5 Composite model

We explored whether the host trait values and evolutionary history represent independent factors for composite model construction by calculating Blomberg's *K* index of phylogenetic signal (Blomberg, Garland, & Ives, 2003). A *K* value of zero indicates random distribution of trait values on the phylogeny, a value of one indicates that trait values are correlated with divergence time according to a Brownian motion model of evolution, and a value greater than one indicates that related species have trait values that are even more similar than expected under Brownian motion (Blomberg et al., 2003). We used the R package Picante (Kembel et al., 2010) to calculate *K* values for each trait and to test against the null hypothesis of random distribution on the phylogeny using 1,000 randomizations of trait values. Ordinal categorical traits (none, low, medium, high) were coded as integers (0, 1, 2, 3) for calculating *K*. We used the same host phylogenetic tree as above, but it was trimmed to include only the species for which trait values were available. Trait values were plotted on the phylogeny using the R package Phylosignal (Keck, Rimet, Bouchez, & Franc, 2016).

We combined the strongly supported submodels (native–novel host divergence time, novel host traits, and native–non‐native insect relatedness; *m* = 1 to 3) predicting risks of high‐impact invasions to estimate the composite risk (*R*) for each of the 221 combinations of conifer hosts (*t*) and conifer specialists (*i*) according to:

(1) $$R_{t,i}=\frac{\sum_{m=1}^{3}\text{logit}\left(\hat{P_{m,t,i}}\right)-\log it\left(P_{m..}\right)}{N_{m}}+\log it\left(P_{\ldots }\right)$$

where $R_{t,i}$ is the estimated probability of high impact (logit units) for the combination of host tree *t* and conifer specialist *i*, $\hat{P_{m,t,i}}$ is the predicted probability of high impact from model *m* for tree *t* and insect *i*, $P_{m..}$ is the proportion of high‐impact incidences for the tree–insect combinations used to parameterize model *m*, $N_{m}$ is the number of models (1–3 depending upon the insect–host combination) yielding predictions for that insect–host pair, and $P_{\ldots }$ is the overall proportion of high‐impact incidences among all 221 insect–host combinations ($P_{\ldots }$ = 0.072).

To evaluate the fit of the predictive model to the observed incidences of high impact, we ranked the predicted probabilities of high impact and allocated them to 10 bins (22 probabilities per bin with 23 in the final bin). The mean probability of each bin was calculated and compared to the observed proportion of high‐impact pairs within the bin.

### 2.6 Model goodness of fit and validation

We calculated *R* ^2^ goodness‐of‐fit metrics to assess the proportion of variability in our dataset explained by each submodel and the composite model. For each submodel, we calculated the Nagelkerke *R* ^2^ (Nagelkerke, 1991) using the fmsb package in R (Nakazawa, 2018). Rather than evaluating predictive ability with data used to train the model, we conducted 10‐fold cross‐validation tests of the submodel on independent data by randomly subsetting the dataset into training (90%) and testing (10%) sets, refitting the model with the training set, making predictions with the testing set, comparing testing set predictions with their known values, replacing the observations, repeating the process nine more times, and averaging the error rate over the 10 iterations (Fushiki, 2011).

Ten‐fold cross‐validation results for each submodel were evaluated using receiver operator characteristic (ROC) plots and area under the curve (AUC) statistics. The AUC score indicates the ability of each submodel to assign a greater likelihood of high impact to an insect–host pair that was actually high impact compared to one that was not (Fielding & Bell, 1997). AUC scores are bounded between 0.00 and 1.00, with a score of 0.50 indicating a model with predictive performance equivalent to random chance and a score of 1.00 indicating perfect predictive ability. Notably, the AUC score for the composite model was not generated with 10‐fold cross‐validation, but with the data used to parameterize it.

## 3 RESULTS

Of the approximately 450 non‐native herbivorous insects currently established in North American forests (Aukema et al., 2010), 58 are conifer specialists, with six historically or currently causing high impacts (Table A1). Only conifer specialists in the insect orders Hymenoptera (i.e., sawflies) and Hemiptera (i.e., adelgids, aphids, and scales) have caused high impact. Conifer hosts were attacked by 1 to 21 non‐native conifer specialists (Table A2), and each insect attacked 1 to 16 novel hosts.

### 3.1 Host phylogeny and insect‐feeding guild predict impact

Divergence time to the most recent common ancestor between the insect's native and novel conifer hosts had strong quadratic relationships to predict the impact for folivores and sap‐feeders. Divergence time was not tested for wood borers, root feeders, and gall makers as none caused high impact.

**Figure 2:**
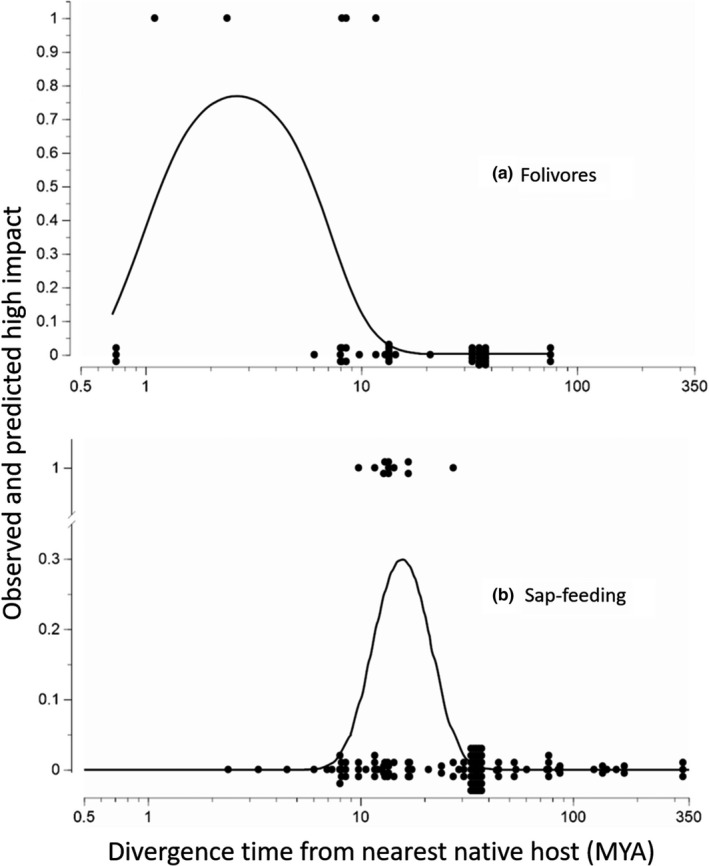
Predicted probability of high impact based on divergence time between native and novel coniferous hosts. For the 49 cases involving folivores (a), the risk of high‐impact invasions was higher [*P*(high impact) ≈ 0.75] with divergence times of 1.5 to 5 mya. For the 131 cases involving sap‐feeding conifer specialists (b), the risk of high impact was greatest [*P*(High Impact) ≈ 0.30] when the North American host tree was of intermediate relatedness to the native host tree (estimated last common ancestor at 10 to 30 mya, zenith at 16 mya). Dots represent observed impact (1 = high impact), and the lines represent predicted impacts based on models. Points have been jittered such that all observations are visible

**Figure 3:**
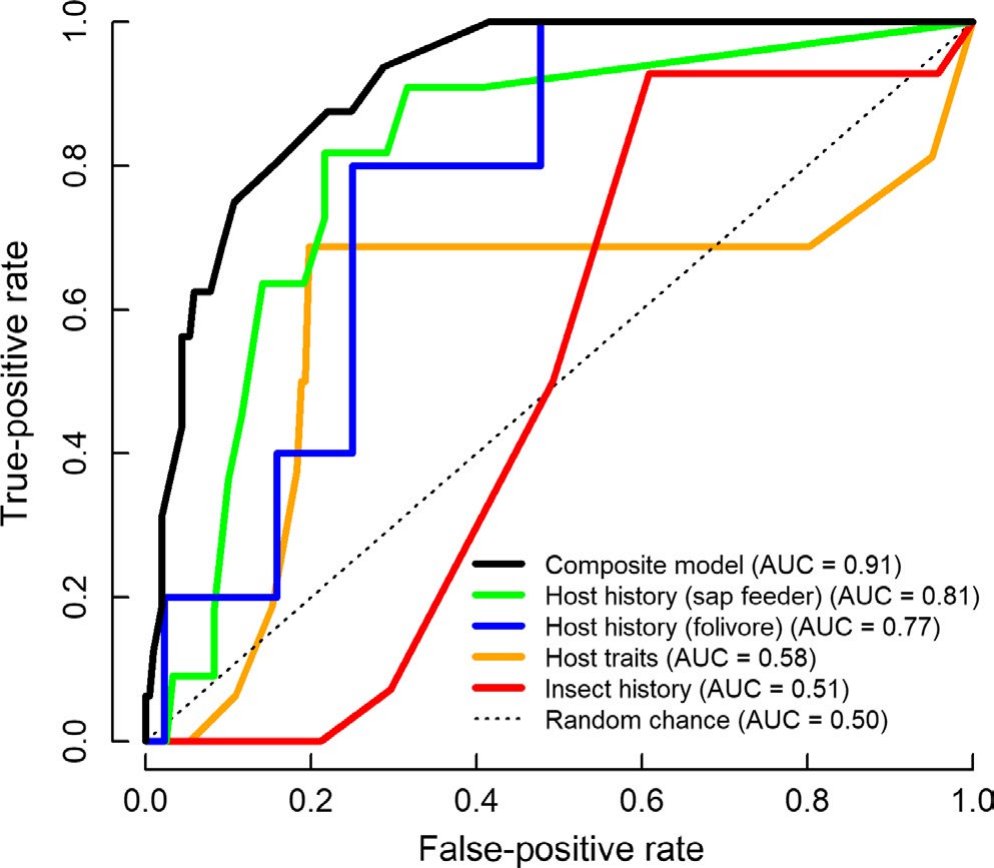
Receiving operator characteristic plot with area under the curve (AUC) statistics for assessing the ability of the model to differentiate high‐impact novel insect–host pairs from non‐high‐impact pairs at different probability thresholds. AUC curves for the four submodels were generated on independent data via 10‐fold cross‐validation, while the AUC curve for the composite model was produced with the full dataset used to parameterize it

The greatest probability of high impact for a folivore conifer specialist was on a novel conifer that diverged from the native conifer host recently (~1.5–5 mya; Figure 2a; Table 5; *p* = .112 and *p* = .072 for divergence time and divergence time^2^, respectively), with probabilities of high impact ranging from .000 to .765 across host divergence times, with the 10th and 90th percentiles encompassing a 12,000‐fold range in probabilities. For native and novel hosts that diverged 2–3 mya, there is a ~76% chance the folivore will cause high impact, but that risk decreases to nearly 0% for hosts more distantly or extremely closely related (Table 6, Figure 2a). Overall, the host evolutionary history model for folivores had moderate predictive performance; *R* ^2^ = 0.43 (Figure 2a) and AUC = 0.77 (Figure 3).

**Table 5:** Parameter estimates for explaining variability in folivores and sap‐feeders for high‐impact insect invasions as a function of time since last common ancestor of the novel North American host and the most closely related native host

Parameter	Estimate	*SE*	*p*‐Value
Folivores			
Intercept	−0.515	1.120	.646
Log10(DivergeTime)	8.073	5.086	.112
Log10(DivergeTime^2^)	−9.495	5.271	.072^b^
Sap‐feeders			
Intercept	−51.824	21.149	.014^a^
Log10(DivergeTime)	84.472	34.739	.014^a^
Log10(DivergeTime^2^)	−35.803	14.182	.012^a^

a Significant at the *α* = 0.05 level

b Significant at the *α* = 0.10 level.

**Table 6:** Comparison of the contributions to risk of high‐impact invasions from individual models and the overall composite model

Predictor model of high‐impact risk	Number of insect–host tree pairs	Variation in risk of high‐impact		
		Standard deviation (logits)	10th−90th percentile (logits)	10th−90th percentile (probabilities)
Host Traits	218	1.03	−4.24 to −1.33	0.014 to 0.209
Host Evolutionary History—Folivores	49	5.36	−10.71 to −0.96	0.000 to 0.277
Host Evolutionary History—Sap‐feeder	131	12.02	−20.64 to −0.95	0.000 to 0.279
Insect Evolutionary History	203	1.03	−4.30 to −2.18	0.013 to 0.102
Composite	221	3.36	−7.96 to −1.70	0.000 to 0.155

Among sap‐feeders, evolutionary divergence time between native and novel hosts had greater predictive power. As with folivores, there was a quadratic relationship between divergence time and impact, but the probability of peak impact occurred at longer divergence times for sap‐feeders (~12–17 mya; Figure 2b; Table 5; *p* = .014 and *p* = .012 for divergence time and divergence time^2^, respectively). The host phylogeny evolutionary submodel for sap‐feeders had an *R* ^2^ value of 0.36 and an AUC score of 0.81 (Figure 3). Predicted probabilities of high impact ranged from infinitesimal (2.85 × 10^−28^) to 0.30 across the range of divergence times for sap‐feeders. The 10th to 90th percentiles had an approximate 257 million‐fold range in probabilities, with a 30% chance that a sap‐feeder will cause high impact on a novel conifer that diverged from the insect's native host about 16 mya; the probability drops to one in over 500 million if the hosts are either closely or distantly related (Figure 2b; Table 6).

### 3.2 Host shade and drought tolerance predict impact

**Figure 4:**
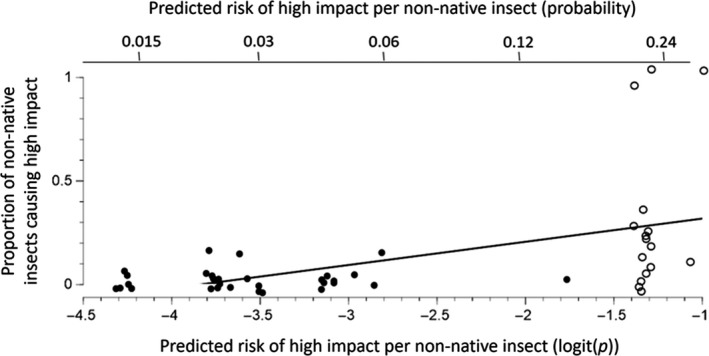
Predicted probability of high impact based on the shade and drought tolerance of the novel host. Comparison of host trait models using multimodel inference indicated that a shade tolerance + drought tolerance model (solid line) received ~ 79% of data support (Table 3). Each point represents one of 49 conifer species that had been challenged by 1 to 21 non‐native conifer‐specialist insects. The *y*‐axis indicates the proportion of non‐native conifer specialists that had high impact on that host species. The *x*‐axis indicates increasing predicted risk from the supported host traits model. Across the range of host traits, the probability of high impact ranged from 0.014 to 0.259, with the cluster of conifer species with the highest risk (open circles) having high shade tolerance (100% of species) and low drought tolerance (88% of species)

Of the nearly 100 conifer species native to North America, 49 were colonized by a non‐native conifer specialist, with 76% colonized by more than one ($\bar{x}$ = 4.44; Table A2). The confidence set predicting high impact as a function of host traits consisted of a single model: shade tolerance + drought tolerance (Tables 3 and 7). Other traits examined that did not influence impact included tree growth rate, wood density, foliage texture, and fire tolerance. The time‐independent (i.e., regardless of time since introduction) predicted probabilities of high impact ranged from 0.014 to 0.259 across hosts. If the novel host was both highly tolerant of shade and had low drought tolerance, life history traits that are highly associated in conifers resulting from fundamental physiological trade‐offs (Rueda, Godoy, & Hawkins, 2017), there was a 20%–26% chance it would experience high impact from a non‐native insect (Figure 4); this included most species of *Abies*, *Picea*, and *Tsuga*. In comparison, novel hosts without high shade and low drought tolerance had as low as a 1.4% chance of experiencing a high‐impact invasion (Figure 4). Independently, the host traits model had a moderate predictive performance with an *R* ^2^ value of 0.19. In addition, a 10‐fold cross‐validation analysis determined an AUC of 0.58 (Figure 3).

**Table 7:** Parameter estimates for the best‐supported model for explaining variability in high‐impact insect invasions as a function of host tree traits

Parameter	Estimate	*SE*	*z*‐Value	*p*‐Value
Intercept	−3.656	1.423	−2.571	.010^a^
Shade tolerance (moderate)	0.634	1.013	0.626	.531
Shade tolerance (high)	2.434	0.816	2.984	.003^a^
Drought tolerance (low)	−0.108	1.297	−0.083	.934
Drought tolerance (moderate)	0.171	1.354	0.126	.899
Drought tolerance (high)	−0.582	1.504	−0.387	.699

Note

In addition to parameter estimates, standard errors (*SE*), *z*‐values, and *p*‐values of the estimates are provided.

a Significant at the *α* = 0.05 level.

### 3.3 Coevolved native insects predict impact

**Figure 5:**
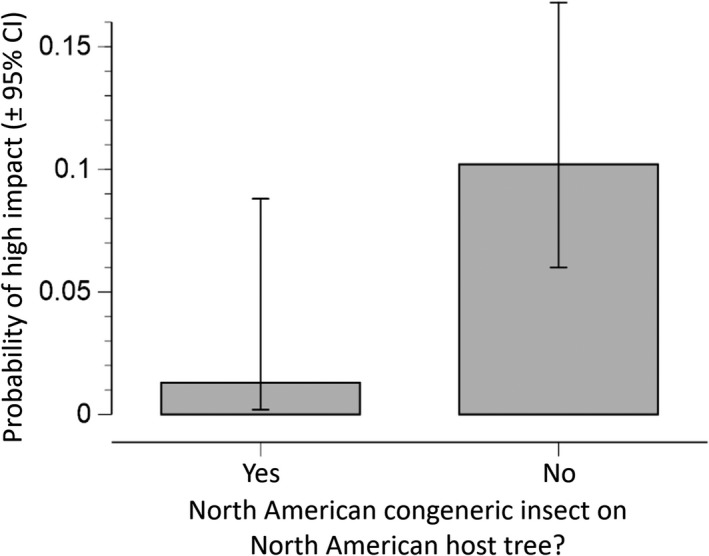
Predicted probability of high impact based on the presence of a North American congener insect on the same conifer species. Model comparisons found that the risk of a non‐native conifer specialist producing high impacts is higher when there is no native (North American) congener that feeds on the shared host [*P*(high impact) = 0.102 vs. 0.013]. This model received ~ 89% of the data support (Table 4). Of the 203 insect–tree pairs, 75 had a congener present on the tree and 128 did not

We evaluated the evolutionary relationship between the non‐native conifer specialist and native North American insects that coevolved with the shared novel conifer host by determining whether they belong to the same genus or family. The presence of a congener feeding on the host significantly decreased the probability that the conifer specialist causes high impact (*p* = .043; Figure 5, Tables 4 and 8). However, the insect evolutionary history model in isolation had relatively poor predictive performance, with an *R* ^2^ value of 0.09 and AUC score of 0.51 (Figure 3).

**Table 8:** Parameter estimates for the best‐supported model for explaining variability in high‐impact insect invasions as a function of the taxonomic relationship between non‐native conifer specialists and their closest North American insect relative on the same host tree species

Parameter	Estimate	*SE*	*z*‐Value	*p*‐Value
Intercept	−2.180	0.293	−7.450	<.001^a^
Shared Genus	−2.124	1.048	−2.026	.043^a^

Note

In addition to parameter estimates, standard errors (*SE*), *z*‐values, and *p*‐values of the estimates are provided.

a Significant at the *α* = 0.05.

### 3.4 Insect life history traits do not predict impact

None of the insect life history traits examined, singly or in combination (Table 2), had predictive value including feeding guild, native region, native pest status, number of native host genera, voltinism, reproductive strategy, fecundity, and/or mechanism of dispersal. Although feeding guild was not a significant predictor of impact directly, we did report quantitatively different models for guilds with respect to the divergence times of the host species. The historical challenge predicting impacts based on insect traits could reflect the lack of variation in traits among high‐ and low‐impact invaders (i.e., univoltinism observed in both groups), further highlighting the importance of factors previously not considered.

### 3.5 Composite model

**Figure 6:**
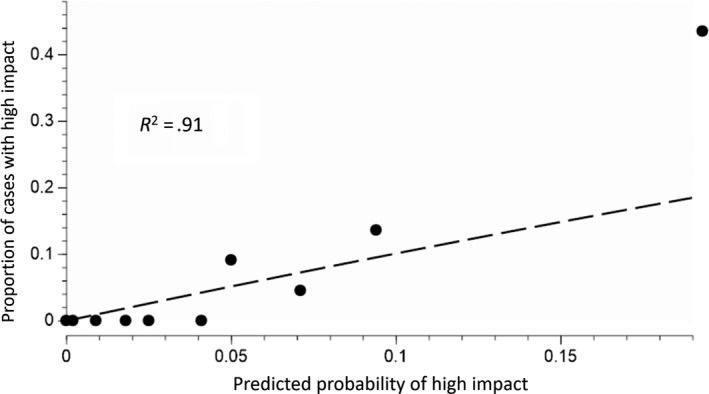
Actual versus predicted risk of high impact based on composite model. Points indicate actual proportions of high impact (*y*‐axis) versus the average predicted risk from the composite model (Equation 1; *x*‐axis). Points represent 10 bins of 22 tree–insect combinations ordered by predicted risk. Dashes indicate the line of equality between observed and predicted cases of high‐impact invasions. *R* ^2^ refers to least squares regression

**Figure A1:**
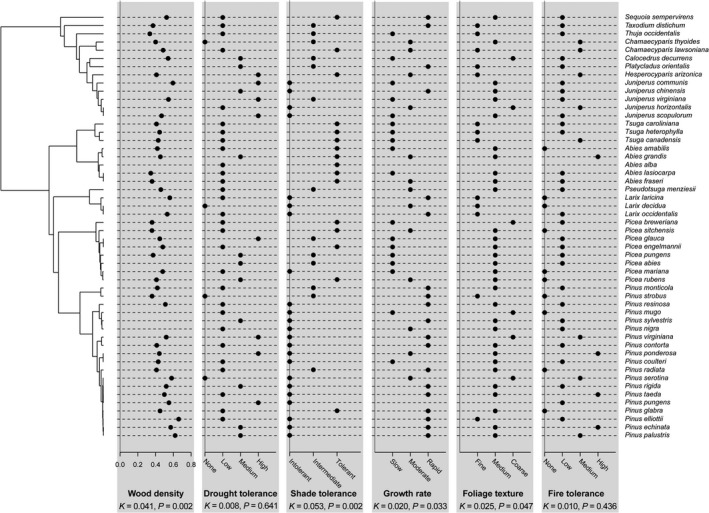
Phylogenetic signal for conifer host traits. Trait values are plotted on the conifer phylogeny that includes only species for which trait values were available. A Blomberg's *K* value of zero indicates random distribution of trait values on the phylogeny, a value of one indicates that trait values are correlated with divergence time. *p*‐Values result from significance tests against the null hypothesis of random distribution of each trait on the phylogeny

The magnitude of correlation between host traits values and divergence time was low for all traits (Blomberg's *K* ranged from 0.008 to 0.053; Figure A1), indicating that the independent host traits and host phylogeny models are not likely to compromise the predictive power of our composite model. The composite model (Equation 1) describes variation in the probability of high impact by non‐native conifer specialists that spans an approximate 443‐fold variation in risk: 0.0003 to 0.1549 for the 10th and 90th percentile of the 221 novel insect–host pairs (Table 6). There was high goodness of fit between predictions of the composite model and observed impacts (*R* ^2^ = 0.91; Figure 6). In addition, the AUC score of 0.91 (Figure 3) indicates that combining submodels increases predictive power. For more than half of the 221 pairs, the predicted risk of high impact was <0.04, with no observed cases of high impact among the 130 pairs with the lowest predicted risks. In contrast, 87.5% of the observed high‐impact cases had a predicted risk above the baseline probability (*p* = .072), providing further support for model fit. The remaining observed high‐impact insect–hosts pairs (*n* = 2) had predicted probabilities above the overall median with an average predicted risk of .048.

Our composite model predicts whether a non‐native conifer specialist will have a one in 6.5 to a one in 2,858 chance of causing high impact on a North American conifer. Although all three submodels contribute to these predictions, the strength of influence varied. By far, the strongest source of variation was the effect of evolutionary divergence time between novel and native hosts on the impact of sap‐feeders and folivores (Figure 2, Table 1). This is particularly insightful as sap‐feeders accounted for a disproportionate share of the non‐native species (57% of conifer specialists and 69% of insect–host pairs). The other submodels had smaller effects on the composite risk of high impact (standard deviation of predicted risk ≈ 1 and changes in relative risk from the 10th to the 90th percentile of 7‐fold to 15‐fold; Table 1).

## 4 DISCUSSION

Only six of the 58 non‐native conifer specialists established in North America historically or currently are causing high impacts: (1) *Adelges piceae*—Balsam woolly adelgid, (2) *Adelges tsugae*—Hemlock woolly adelgid, (3) *Elatobium abietinum—*Green spruce aphid, (4) *Gilpinia hercyniae*—European spruce sawfly, (5) *Matsucoccus matsumurae*—Red pine scale, and (6) *Pristiphora erichsonii*—Larch sawfly. All high‐impact, non‐native conifer specialists in North America belong to the orders Hemiptera or Hymenoptera.

The greatest power of our composite model for predicting high impact came from the submodels related to evolutionary history between native and novel hosts. Intimacy of host association has been proposed as a significant factor affecting evolutionary responses of plants to herbivory (Mattson, Lawrence, Haack, Herms, & Charles, 1988; Walling, 2000). This may help explain why the evolutionary divergence time between native and novel hosts at which peak impact occurred was greater for sap‐feeders than for folivores. Sap‐feeders are considered to have a more intimate association with their hosts than folivores because they feed with their mouthparts embedded within specific plant tissues and cells, often for long periods of time (Walling, 2000). This can create a greater opportunity for the exchange of highly specific cues and molecular signals that can elicit precisely targeted host defenses and insect responses (Stuart, 2015; Walling, 2000; Yates & Michel, 2018; Züst & Agrawal, 2016). Indeed, examples of coevolutionary deme selection in which insects adapt to individual host plants derive almost exclusively from sap‐feeders (Hanks & Denno, 1993).

A meta‐analysis found that sap‐feeders can decrease the growth, photosynthesis, and reproduction of conifers (Zvereva, Lanta, & Kozlov, 2010), which should select for targeted defenses. Novel conifer hosts that recently diverged from the native host of a non‐native conifer specialist may retain defenses evolved during past interaction with the herbivore, thus contributing to lower impact of non‐native sap‐feeders on the novel host. As host divergence times increase, herbivore resistance and/or tolerance of the novel host may relax, especially if there are costly physiological and ecological trade‐offs associated with maintaining these traits (Herms & Mattson, 1992). This would increase the probability that an invading sap‐feeder will have high impact on a novel host. As evolutionary divergence time between the native and novel hosts continues to increase, the conifers may have diverged genetically and physiologically to the point that sap‐feeders have limited ability to recognize and subsequently impact the novel host.

Conversely, it has been hypothesized that folivores are less likely than sap‐feeders to select for highly specific host recognition and defense responses because they generally have a less intimate relationship with their host (Mattson et al., 1988; Walling, 2000). Host pairs that diverged very recently (<1 mya) may retain effective defenses in the absence of herbivory until they are selected against because their costs outweigh their benefits in the absence of herbivory (Herms & Mattson, 1992). Consequently, non‐native folivores may recognize, consume, and thus severely impact poorly defended novel hosts as they continue to diverge from the native host if they retain enough similarity traits that facilitate host finding and acceptance. As the time of evolutionary divergence between the native and novel hosts becomes more distant, traits affecting host utilization should increasingly diverge, decreasing the ability of non‐native folivores to impact or even recognize novel hosts.

Shade and drought tolerance were the only host traits we examined that predicted degree of host impact. Availability of light and water are major selection pressures shaping the life history of conifers (Rueda et al., 2017) and optimal evolution of plant defense strategies (Coley, Bryant, & Chapin, 1985; Herms & Mattson, 1992). Shade tolerance is predicted to be associated with strong defense because it may be more difficult to compensate for tissues lost to herbivory in light‐limited environments due to low rates of net photosynthesis (Coley et al., 1985; Strauss & Agrawal, 1999). Indeed, shade‐tolerant plants have been found to be better defended and experience less herbivory than shade‐intolerant plants (Coley, 1983). Yet, our results found that novel shade‐tolerant/drought‐intolerant conifers were more likely to experience high impacts from non‐native insects (Figure 4). This could indicate that if shade‐tolerant conifers have limited ability to tolerate herbivory, then the impact of non‐native specialist insects preadapted to overcoming host defenses may be high. We are not aware of studies of interspecific variation in herbivore tolerance of conifers as it relates to their shade tolerance. Within a species, however, shade has been shown to decrease the ability of conifers to compensate for herbivory (Baraza, Zamora, & Hódar, 2010; Saunders & Puettmann, 1999).

The presence of a native congener feeding on the novel host decreased the probability that a conifer specialist caused high impact, perhaps due to biotic resistance resulting from one or a combination of factors (Nunez‐Mir et al., 2017). For example, host defense and tolerance traits selected in response to the native congener could be effective against the closely related non‐native conifer specialist (allopatric resistance; Harris, 1975). In addition, the non‐native conifer specialist could be susceptible to specialist and/or generalist natural enemies of the congener (Carlsson, Sarnelle, & Strayer, 2009). Finally, the native congener could be better adapted to a shared niche and thus be a stronger competitor than the evolutionarily naïve non‐native conifer specialist (Paini, Funderburk, & Reitz, 2008).

## 5 CONCLUSIONS

Understanding what factors drive the impact of non‐native species is a central goal in invasion biology, yet hypotheses have remained largely untested. Our work offers quantitative insight into the role that evolutionary history plays in predicting which non‐native insects will cause high impacts. Specifically, we have demonstrated that the probability of high impact can be predicted from host plant traits, the divergence time between the insect's native and novel hosts, and the presence or absence of a coevolved congener feeding on the same host. Importantly, we concluded that traits of the invading insect that we examined, except for the indirect effect of feeding guild, cannot be used to predict the insect's impact in its non‐native range. Rather, the three categories of factors important in determining the host impact of non‐native conifer specialists all directly, or through an interaction, involve the novel host. These findings suggest that the invaded host or invaded community, including the history of evolutionary relationships among community members, is more important for predicting impact than life history traits of the invading insect.

This model can also be used to quantify, with assigned statistical confidence, the probability that conifer specialists will cause high impacts should they establish in North America. From a practical perspective, the model can be used to assess risk posed by non‐native insects and allocate scarce management resources. It is worth noting that the model created is only as strong as the data available, which are reasonably complete for the most economically significant insect–host pairs. However, false positives or negatives will impact the probability of risk for variables where data are incomplete, which, for example, is probable for insect–host lists in both the native and introduced range (e.g., Wagner & Todd, 2016). A positive attribute of the structure of the composite model (Equation 1) is that it is an adaptive model that lends itself to continuing evaluation and improvement as data accumulate. It is an unfortunate certainty that non‐native conifer specialists will continue to establish in North America, with each new introduction increasing the pool of novel insect–host interactions that can be evaluated. Furthermore, advances in the understanding of invasion ecology and plant–herbivore interactions will inform hypotheses about causes of high‐impact invasions that we did not evaluate. Given our findings, evolutionary history is central to understanding and predicting interactions between non‐native insects and their novel hosts.

## CONFLICT OF INTEREST

The authors declare no conflict of interest.

## AUTHOR'S CONTRIBUTIONS

TDM, KAT, DAH, and PCT conceived the project. All authors contributed to the study design; AMM, ANS, NPH, and RAH collected the study data; DRU, MPA, PCT, AMM, and CRA analyzed the data; all authors provided feedback on interpretation of results and wrote/edited the manuscript.

## Data Availability

Data supporting the results are archived in the US Geological Survey ScienceBase‐Catalog (Mech, Havill, Schulz, & Thomas, 2019) and will be publicly available after March 2020.

## APPENDIX 1

**Table A1:** Information pertaining to non‐native conifer specialists in North America

Conifer‐specialist species	Insect order	Insect family	Native range	Feeding guild	Impact number	High impact
*Acantholyda erythrocephala*	Hymenoptera	Pamphiliidae	Europe	Folivore	4	0
*Adelges abietis*	Hemiptera	Adelgidae	Europe	Gall	2	0
*Adelges laricis*	Hemiptera	Adelgidae	Europe	Sap	2	0
*Adelges piceae*	Hemiptera	Adelgidae	Europe	Sap	9	1
*Adelges tsugae*	Hemiptera	Adelgidae	Asia	Sap	9	1
*Aethes rutilana*	Lepidoptera	Cochylidae	Europe	Folivore	2	0
*Aspidiotus cryptomeriae*	Hemiptera	Diaspididae	Asia	Sap	2	0
*Atractotomus magnicornis*	Hemiptera	Miridae	Europe	Sap	1	0
*Brachyderes incanus*	Coleoptera	Curculionidae	Europe	Root	5	0
*Callidiellum rufipenne*	Coleoptera	Cerambycidae	Asia	Wood	2	0
*Camptozygum aequale*	Hemiptera	Miridae	Europe	Sap	1	0
*Carulaspis juniperi*	Hemiptera	Diaspididae	Europe	Sap	5	0
*Carulaspis minima*	Hemiptera	Diaspididae	Europe	Sap	5	0
*Cinara cupressi*	Hemiptera	Aphididae	Europe	Sap	2	0
*Cinara pilicornis*	Hemiptera	Aphididae	Eurasia	Sap	1	0
*Cinara pinea*	Hemiptera	Aphididae	Eurasia	Sap	1	0
*Cinara tujafilina*	Hemiptera	Aphididae	Asia	Sap	2	0
*Coleophora laricella*	Lepidoptera	Coleophoridae	Europe	Folivore	5	0
*Contarinia baeri*	Diptera	Cecidomyiidae	Europe	Folivore	2	0
*Crypturgus pusillus*	Coleoptera	Curculionidae	Eurasia	Wood	1	0
*Dichomeris marginella*	Lepidoptera	Gelechiidae	Europe	Folivore	2	0
*Dichrooscytus rufipennis*	Hemiptera	Miridae	Europe	Sap	1	0
*Diprion similis*	Hymenoptera	Diprionidae	Eurasia	Folivore	6	0
*Dynaspidiotus pseudomeyeri*	Hemiptera	Diaspididae	Asia	Sap	1	0
*Dynaspidiotus tsugae*	Hemiptera	Diaspididae	Asia	Sap	2	0
*Elatobium abietinum*	Hemiptera	Aphididae	Europe	Sap	6	1
*Epinotia nanana*	Lepidoptera	Tortricidae	Europe	Folivore	2	0
*Eulachnus agilis*	Hemiptera	Aphididae	Europe	Sap	2	0
*Eulachnus brevipilosus*	Hemiptera	Aphididae	Europe	Sap	2	0
*Eulachnus rileyi*	Hemiptera	Aphididae	Europe	Sap	2	0
*Exoteleia dodecella*	Lepidoptera	Gelechiidae	Europe	Folivore	2	0
*Fiorinia externa*	Hemiptera	Diaspididae	Asia	Sap	5	0
*Gilpinia frutetorum*	Hymenoptera	Diprionidae	Eurasia	Folivore	2	0
*Gilpinia hercyniae*	Hymenoptera	Diprionidae	Europe	Folivore	6	1
*Grypotes puncticollis*	Hemiptera	Cicadellidae	Europe	Sap	1	0
*Hylastes opacus*	Coleoptera	Curculionidae	Eurasia	Wood	3	0
*Hylurgops palliatus*	Coleoptera	Curculionidae	Eurasia	Wood	3	0
*Hylurgus ligniperda*	Coleoptera	Curculionidae	Eurasia	Wood	2	0
*Matsucoccus matsumurae*	Hemiptera	Matsucoccidae	Asia	Sap	7	1
*Neodiprion sertifer*	Hymenoptera	Diprionidae	Eurasia	Folivore	2	0
*Ocnerostoma piniariella*	Lepidoptera	Yponomeutidae	Europe	Folivore	2	0
*Orthotomicus erosus*	Coleoptera	Curculionidae	Eurasia	Wood	1	0
*Phoenicocoris dissimilis*	Hemiptera	Miridae	Europe	Sap	1	0
*Phyllobius intrusus*	Coleoptera	Curculionidae	Asia	Root	2	0
*Physokermes hemicryphus*	Hemiptera	Coccidae	Europe	Sap	2	0
*Pinalitus rubricatus*	Hemiptera	Miridae	Europe	Sap	1	0
*Pineus boerneri*	Hemiptera	Adelgidae	Asia	Sap	3	0
*Pineus pineoides*	Hemiptera	Adelgidae	Europe	Sap	1	0
*Pineus pini*	Hemiptera	Adelgidae	Europe	Sap	1	0
*Pityogenes bidentatus*	Coleoptera	Curculionidae	Eurasia	Wood	1	0
*Plagiognathus vitellinus*	Hemiptera	Miridae	Eurasia	Sap	1	0
*Pristiphora erichsonii*	Hymenoptera	Tenthredinidae	Eurasia	Folivore	6	1
*Rhyacionia buoliana*	Lepidoptera	Tortricidae	Europe	Folivore	2	0
*Schizolachnus pineti*	Hemiptera	Aphididae	Europe	Sap	1	0
*Sirex noctilio*	Hymenoptera	Siricidae	Eurasia	Wood	5	0
*Spilonota lariciana*	Lepidoptera	Tortricidae	Europe	Folivore	1	0
*Thera juniperata*	Lepidoptera	Geometridae	Europe	Folivore	2	0
*Tomicus piniperda*	Coleoptera	Curculionidae	Eurasia	Wood	3	0

Note

High‐impact binomial value: 1 = yes, 0 = no.

**Table A2:** North American conifer hosts fed on by non‐native conifer‐specialist insects

North American conifer host species	Number of non‐native conifer specialists	Highest impact number	High impact
*Abies amabilis*	1	6	1
*Abies balsamea*	6	8	1
*Abies fraseri*	4	9	1
*Abies grandis*	1	6	1
*Abies lasiocarpa*	1	8	1
*Calocedrus decurrens*	2	2	0
*Chamaecyparis lawsoniana*	2	2	0
*Chamaecyparis thyoides*	4	5	0
*Cupressus arizonica*	1	2	0
*Hesperocyparis goveniana*	1	2	0
*Hesperocyparis macrocarpa*	2	2	0
*Juniperus communis*	8	5	0
*Juniperus horizontalis*	2	2	0
*Juniperus scopulorum*	1	2	0
*Juniperus virginiana*	9	5	0
*Larix laricina*	8	6	1
*Larix lyalii*	1	2	0
*Larix occidentalis*	2	5	0
*Picea breweriana*	2	1	0
*Picea engelmanni*	4	6	1
*Picea glauca*	10	6	1
*Picea mariana*	5	6	1
*Picea pungens*	9	6	1
*Picea rubens*	7	6	1
*Picea sitchensis*	4	6	1
*Pinus banksiana*	11	3	0
*Pinus contorta*	7	2	0
*Pinus coulteri*	2	2	0
*Pinus echinata*	3	2	0
*Pinus elliotti*	1	2	0
*Pinus glabra*	1	2	0
*Pinus monticola*	2	2	0
*Pinus palustris*	1	2	0
*Pinus ponderosa*	8	2	0
*Pinus pungens*	2	2	0
*Pinus radiata*	6	2	0
*Pinus resinosa*	21	7	1
*Pinus rigida*	7	2	0
*Pinus serotina*	1	2	0
*Pinus strobus*	17	6	1
*Pinus taeda*	3	2	0
*Pinus virginiana*	5	2	0
*Pseudotsuga menziesii*	5	2	0
*Sequoia sempervirens*	2	2	0
*Taxodium distichum*	1	2	0
*Thuja occidentalis*	8	5	0
*Tsuga canadensis*	6	8	1
*Tsuga caroliniana*	3	9	1
*Tsuga heterophylla*	1	1	0

Note

High‐impact binomial value: 1 = yes; 0 = no.
